# Flux-Reducing Tendency of Pd-Based Membranes Employed in Butane Dehydrogenation Processes

**DOI:** 10.3390/membranes10100291

**Published:** 2020-10-16

**Authors:** Thijs A. Peters, Marit Stange, Rune Bredesen

**Affiliations:** SINTEF Industry, P.O. Box 124 Blindern, N-0314 Oslo, Norway; marit.stange@sintef.no (M.S.); rune.bredesen@sintef.no (R.B.)

**Keywords:** palladium membrane, dehydrogenation, butylene, flux-reducing tendency

## Abstract

We report on the effect of butane and butylene on hydrogen permeation through thin state-of-the-art Pd–Ag alloy membranes. A wide range of operating conditions, such as temperature (200–450 °C) and H_2_/butylene (or butane) ratio (0.5–3), on the flux-reducing tendency were investigated. In addition, the behavior of membrane performance during prolonged exposure to butylene was evaluated. In the presence of butane, the flux-reducing tendency was found to be limited up to the maximum temperature investigated, 450 °C. Compared to butane, the flux-reducing tendency in the presence of butylene was severe. At 400 °C and 20% butylene, the flux decreases by ~85% after 3 h of exposure but depends on temperature and the H_2_/butylene ratio. In terms of operating temperature, an optimal performance was found at 250–300 °C with respect to obtaining the highest absolute hydrogen flux in the presence of butylene. At lower temperatures, the competitive adsorption of butylene over hydrogen accounts for a large initial flux penalty.

## 1. Introduction

Alkene production by the catalytic dehydrogenation (DH) of light alkanes is an alternative to conventional heavy hydrocarbon cracking [1,2]. Dehydrogenation is an endothermic equilibrium-limited reaction and is typically performed at elevated temperatures at close to atmospheric pressure. Even at 500 °C, the thermodynamic equilibrium conversion for propane dehydrogenation is less than 20%. Further, the high operating temperature results in large amounts of carbon deposition on the catalyst, which implies the need for a periodic regeneration of the catalytic bed, leading to a complex plant design with multiple reactors, up to 5–8, in parallel, operating at temperatures between 500 and 600 °C [2], which is only feasible at large scale [3,4]. Due to the removal of hydrogen from the reaction, membrane reactors have the potential to provide the same conversion and yield of a conventional process while operating at milder conditions [5,6,7,8,9,10,11,12,13,14,15,16,17,18,19,20,21,22,23,24]. This would potentially reduce the level of catalyst deactivation observed in the conventional dehydrogenation of light alkanes. Additionally, downstream separations are simplified, as most of the hydrogen is separated in situ. It should be noted though that appropriate geometrical designs and operating conditions of a packed-bed membrane reactor are key to use the effect of hydrogen permeable membranes on actually obtaining the intended shift of the equilibrium towards more alkylenes [25].

Among all H_2_-selective membrane types, Pd-based membranes show an optimum match with the operating conditions typically targeted in a membrane-enhanced dehydrogenation process that is potentially operated between 300 and 500 °C [26,27]. One of the most investigated challenges for Pd-based membranes is that they are, to various degrees, prone to poisoning by for example CO and other surface co-adsorbates, leading to a reduced H_2_ flux through coking [28,29,30,31,32,33,34,35,36,37]. Also in the case of dehydrogenation of light alkenes, it was observed that the obtained H_2_ flux decreases over time [6,13,15,38,39]. The most common explanation for the flux decrease is coke formation accumulating on the membrane surface, thereby suppressing the H_2_ permeation. In a recent study, the evolution of various surface and bulk carbon species derived from propylene and their influence on the hydrogen flux was investigated. Results indicate that there are at least four different carbon species that are deposited onto the Pd foil during C_3_H_6_ exposure [40]. Primarily, the surface-adsorbed C_x_H_y_ species are responsible for the H_2_ flux inhibition, but it is shown that their structure evolves to become subsurface or even bulk carbon species with increasing exposure time, concentration, and temperature. At temperatures below 300–400 °C, bulk C species are not formed due to the large activation barrier for C diffusion in the bulk of Pd. The results presented in [40] agree well with our previous work investigating the effect of propylene on hydrogen permeation through thin state-of-the-art Pd–Ag and Pd–Cu alloy membranes [39]. In that work, with the specific aim to investigate operating conditions that result in a negligible flux reduction, a wide range of operating conditions, such as temperature and H_2_/propylene ratio, were investigated. It was shown that coking could be limited at lower operating temperature, and a decrease to at least 300 °C or, preferably, to 250 °C is required to achieve sufficiently stable membrane operation in the conditions observed in a non-integrated sequential reactor-membrane process design [41]. In the sequential reactor-membrane process, a stable membrane performance was obtained at 200 °C at hydrogen recover factor (HRF) values varying from 38 to 50% [39].

In the present work, we report a thorough investigation of the effect of butane and butylene on hydrogen permeation through thin Pd–Ag alloy membranes. The effects of a wide range of operating conditions, such as temperature and H_2_/butylene/butane ratio, on the flux-reducing tendency were investigated. In addition, the behavior of membrane performance during prolonged exposure to butylene was evaluated. Finally, the long-term stability of the membrane itself was investigated and characterized through a post-process characterization of applied membranes. As in previous works, experiments were conducted using thin state-of-the-art self-standing Pd–Ag foils integrated in a microchannel-configured module. This configuration is very well suited for the investigation of surface inhibition effects caused by co-existing species in the gaseous feed under different operating conditions in the absence of any flux-limiting factors such as concentration polarization [42,43].

## 2. Materials and Methods

### 2.1. Pd Alloy Module Preparation

Pd alloy films were prepared by magnetron sputtering from a Pd_77_Ag_23_ target onto a silicon substrate, and subsequently integrated in a microchannel configuration to allow for hydrogen permeation measurements. Membrane films with a nominal thickness of 10 micron were applied in the current work. The microchannel system applied in this work consisted of a stainless steel feed channel section with thirteen parallel channels machined with dimensions 0.2 × 0.2 × 13 mm^3^, and is similar to the module applied in our previous work on propylene [39,44]. On the permeate side of the membrane, a perforated steel support plate was mounted. In total, the thirteen channels provided for a 0.449 cm^2^ active membrane area. The Pd_77_Ag_23_ film-loaded membrane module was then connected to the gas system to allow for flux measurements. Two membrane modules were applied during the investigation.

### 2.2. Gas Permeation Measurements

#### 2.2.1. H_2_ Permeation Experiments

The H_2_ permeation system reported previously in [39,45,46] was used for the permeation and exposure experiments. When heating up the module, the membrane was flushed with N_2_ (99.999%) and Ar (99.999%) on the feed and permeate side, respectively, until 300 °C was reached. Then H_2_ (99.995%) was introduced at the feed side. H_2_ flux measurements were performed in the temperature range 450–300 °C. The total feed flow rate applied for all measurements equals 500 NmL·min^−1^. Based on previous experience, this feed flow rate is sufficient to suppress the effect of H_2_ depletion along the microchannel system. A sweep flow of 250 or 500 NmL·min^−1^ of Ar (99.999%) was applied at the permeate side. The H_2_ flux was calculated from the measured hydrogen concentration in the permeate using the calibrated flow of Ar sweep gas. For this, the permeate composition was measured by means of a micro GC (µGC, Agilent 490, Santa Clara, CA, USA). The hydrogen permeance was calculated by dividing the hydrogen flux by the hydrogen partial pressure difference $\left(P_{H_{2}^{ret}}^{0.5}-P_{H_{2}^{perm}}^{0.5}\right)$ over the membrane. The nominal membrane thickness of 10 micron was used in the calculation of the H_2_ permeability.

#### 2.2.2. H_2_ Permeation Experiments

The effect of butane and butylene on the H_2_ flux was investigated applying feed mixtures originally consisting of H_2_ in N_2_. Butane or butylene were then introduced by simply exchanging a part of N_2_. In this way, the feed H_2_ concentration was kept constant. In between different exposure studies, the membrane was regenerated applying a heat treatment in air (HTA). The HTA of the membrane surface recovers the H_2_ flux to its original value [38,40]. The HTA treatment was carried out by opening the module connections after flushing with nitrogen and argon, allowing air to enter the module at 400 °C for 1 h. Afterwards, the connections were re-connected followed by sufficient flushing with nitrogen and argon prior to H_2_ introduction.

#### 2.2.3. Post-Process Characterization

The plan-view and cross-section microstructure of the employed membrane film were characterized after the permeation experiments by scanning electron microscopy (SEM) using secondary electrons (SE) or backscattered electrons (BSE), with an FEI 650 NOVA NanoSEM instrument (FEI Company, Hillsboro, OR, USA) combined with energy dispersive spectroscopy (EDS) (X-MAX50, Oxford Instruments, Abingdon, Oxfordshire, UK).

## 3. Results and Discussion

### 3.1. H_2_ Permeation Properties of Employed Pd-Based Membranes

**Figure 1:**
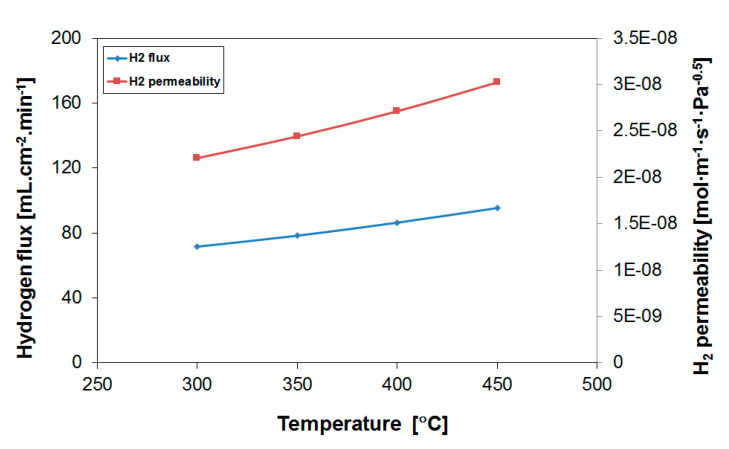
Hydrogen flux and permeability as a function of temperature for the Pd_77_Ag_23_ alloy membrane. Feed: 80% H_2_ in N_2_.

H_2_ permeation measurements through the 10 micron-thick Pd_77_Ag_23_ membranes were performed between 300 and 450 °C applying a feed mixture of H_2_:N_2_ = 80:20 at atmospheric pressure. The results for the H_2_ flux and calculated permeability can be seen in Figure 1 for the first module employed in the experiments.

A hydrogen flux of 95.4 mL·cm^−2^·min^−1^ was obtained at a temperature of 450 °C. Given the experimental conditions, this corresponds to a hydrogen permeability of 3.0 × 10^−8^ mol·m^−1^·s^−1^·Pa^−0.5^. The flux and permeability values obtained are shown in Table 1 as a function of the operation temperature. In the calculation of the permeability, the *n*-value was forced to 0.5. The activation energy of the permeation equals 7.2 kJ·mol^−1^, close to reported values for Pd_77_Ag_23_ membranes [47]. Previous reported values for Pd_77_Ag_23_ membranes prepared by the same method are in the range of 8 × 10^−9^–3.2 × 10^−8^ mol·m^−1^·s^−1^·Pa^−0.5^ (400 °C) for membranes with a thickness between 1.4 and 10 µm [45,46,47,48], agreeing well with current results. 

### 3.2. Exposure to Butane

**Figure 2:**
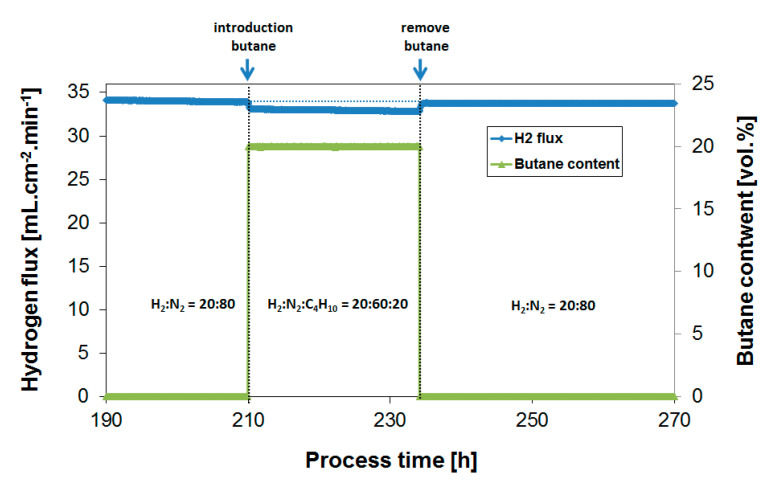
H_2_ flux during butane introduction (20%) and overnight exposure to a feed mixture of H_2_:N_2_:C_4_H_10_ = 20:60:20. Temperature = 400 °C.

The effect of butane on the H_2_ flux performance was investigated applying a feed mixture of H_2_:C_4_H_10_:N_2_ = 20:20:60 in order to simulate the butane dehydrogenation process. As in previous experiments with respect to propane [39], initial experiments were performed at 400 °C. Due to the low butane vapor pressure at room temperature, 1.6 bar, the exposure was performed at atmospheric pressure. Figure 2 shows the H_2_ flux during the introduction (process time of 210 h) and in presence of 20% butane, respectively. Note that the obtained H_2_ flux value is lower compared to the initial results presented in Figure 1 because of the lower H_2_ feed content.

**Figure 3:**
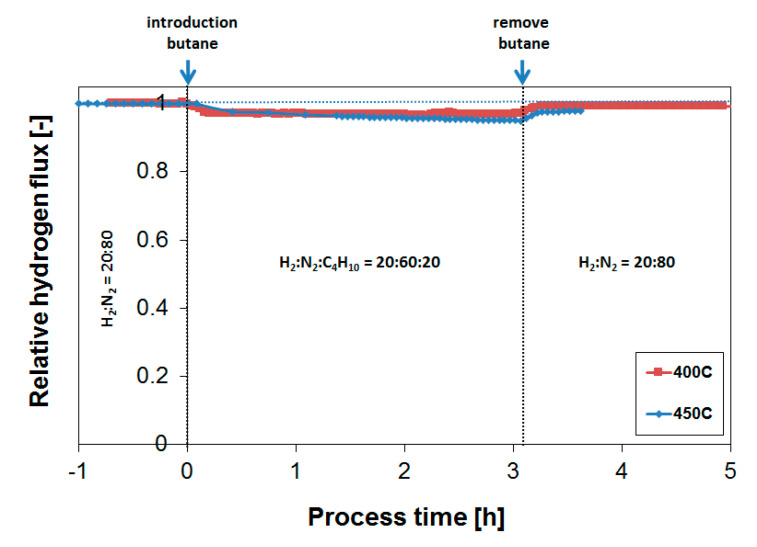
Effect of temperature on the obtained relative H_2_ flux during butane exposure applying a feed mixture of H_2_:C_4_H_10_:N_2_ = 20:20:60.

The butane introduction resulted in a H_2_ flux decline of approximately 2–3%. The slight decrease in flux is presumably explained by a small mismatch in the applied flows, resulting in a minor decrease in H_2_ feed content upon butane introduction. Most importantly, the continuous exposure for 24 h to a feed of H_2_:N_2_:C_4_H_10_ = 20:60:20 resulted in a relatively constant H_2_ flux, showing that the tendency for coke formation in the presence of butane is minimal. The removal of butane (process time of 234 h) resulted as well in a complete flux recovery, suggesting that the observed inhibition was governed by a reversible adsorption process, and not by irreversible coke formation that was expected to be removed much slower from the surface. Subsequently, the butane effect was investigated at 450 °C. Figure 3 summarizes obtained H_2_ flux inhibition curves as a function of temperature plotted as relative H_2_ flux values at 400 and 450 °C. The relative H_2_ flux corresponds to the measured H_2_ flux in the presence of butane normalized by the H_2_ flux obtained before the butane introduction under otherwise equal conditions of temperature, absolute feed pressure, hydrogen partial pressure and total feed flow rate. 

Even though the flux decline was slightly accelerated at higher temperatures, the flux reducing coke formation tendency was still relatively limited at 450 °C. Comparing this limited flux decrease in the presence of butane with previous results for propane [39] shows that the H_2_ flux reducing tendency for butane was less compared to propane. A similar membrane operated at 450 °C in a mixture of H_2_:N_2_:C_3_H_8_ = 30:20:50 shows a gradual H_2_ flux decrease to a value of approximately 65% of the original H_2_ flux after 18 h. It should be noted that the ratio between H_2_ and the hydrocarbon determines the extent of coke formation. In that respect, a direct comparison is not directly possible, as [39] applied a H_2_ to propane ratio of 0.6. It should also be noted that the operating pressure is not the same either—1 bar versus 4 bar—even though this seemingly does not affect coke formation tendency. 

### 3.3. Parametric Study of H_2_ Flux Inhibition During Butylene Exposure

**Figure 4:**
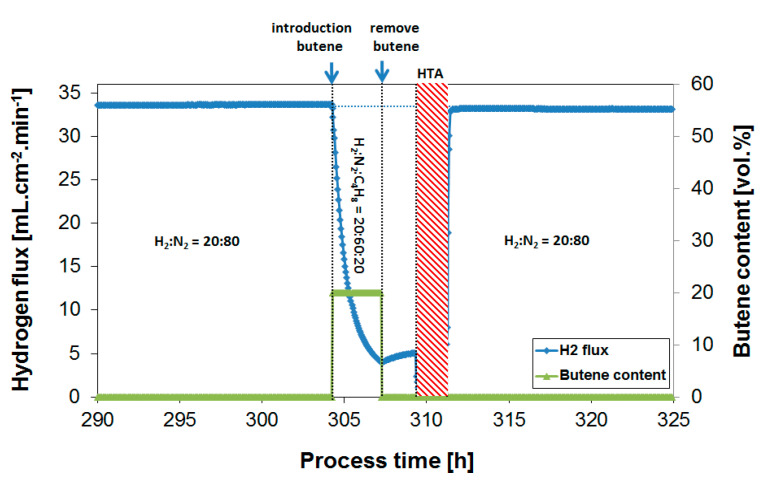
H_2_ flux during butylene exposure (20%) to a feed mixture of H_2_:N_2_:C_4_H_10_ = 20:60:20, and subsequent air treatment to regenerate the membrane performance. Temperature = 400 °C.

**Figure 5:**
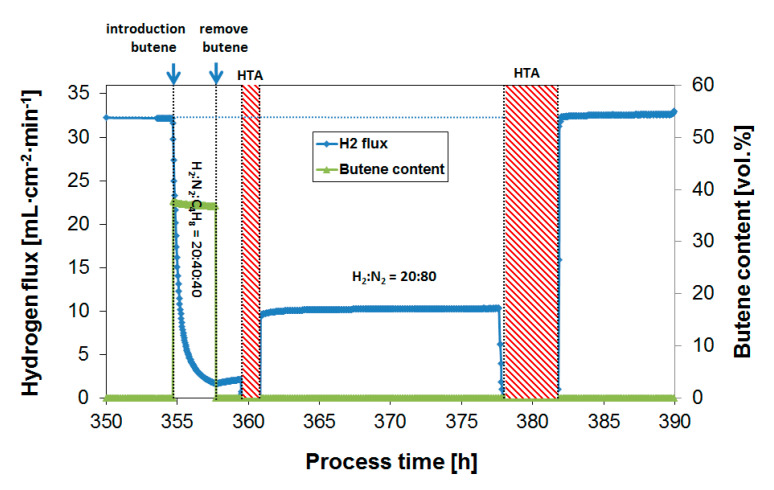
H_2_ flux during butylene exposure (40%) to a feed mixture of H_2_:N_2_:C_4_H_10_ = 20:40:40, and subsequent air treatments to regenerate the membrane performance. Temperature = 400 °C.

The effect of butylene on the H_2_ flux performance was investigated at varying H_2_/butylene ratios. For the initial experiments, a temperature of 400 °C was chosen, similar to the butane experiments described in Section 3.2. Figure 4 and Figure 5 show the H_2_ flux during exposure to a feed containing 20 or 40% butylene, respectively, at a H_2_ content of 20%. It was previously shown that the extent of coke formation is not determined by the absolute propylene concentration, but by the ratio between H_2_ and propylene [39,40].

**Figure 6:**
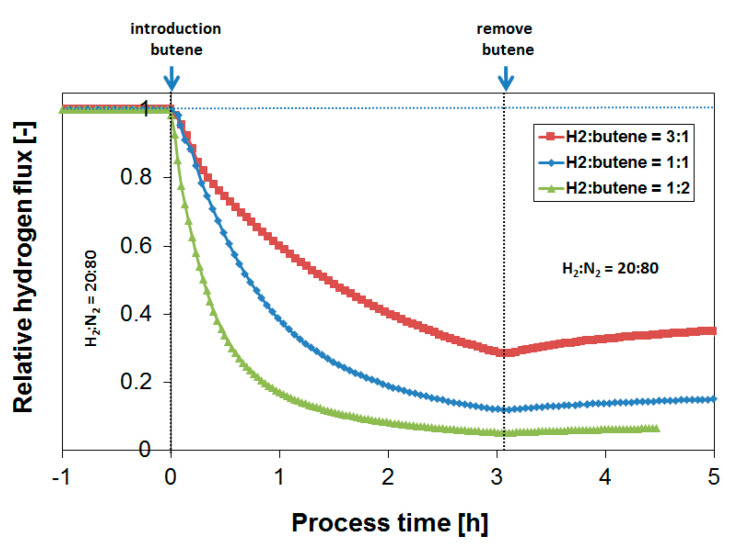
Effect of the H_2_/butylene ratio in the membrane feed on the obtained relative H_2_ flux after the butylene introduction. Temperature = 400 °C.

**Figure 7:**
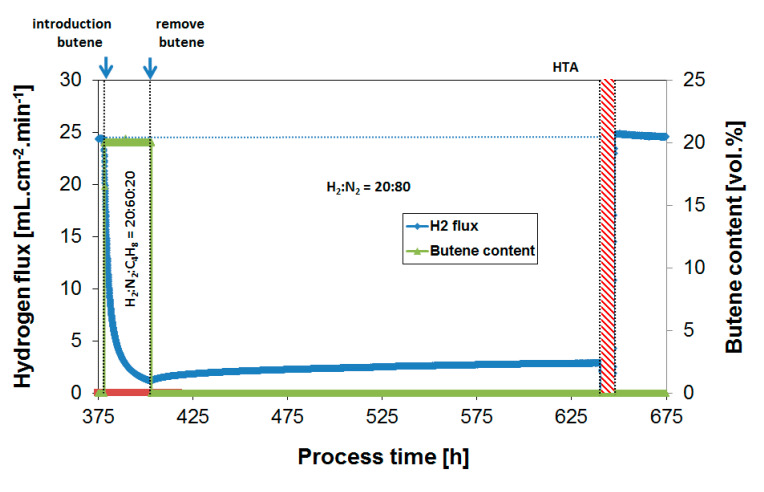
H_2_ flux during continuous recovery after butylene exposure (20%) to a feed mixture of H_2_:N_2_:C_4_H_10_ = 20:60:20. Subsequent air treatment to regenerate the membrane performance. Temperature = 350 °C.

Upon butylene exposure, the H_2_ flux rapidly decreases; in the case of 20% butylene, the flux dropped by ~85% after 3 h of exposure compared to the H_2_ flux prior to the butylene exposure. This is directly related to the gradual generation of surface-adsorbed C_x_H_y_ species or coke formation on the membrane leading to a deactivation of the membrane surface. The large difference in coking tendency between alkanes and alkylenes was reported in an early study by Collins et al. [15] and in our previous work applying the same methodology [39]. This difference is explained by the much larger alkylene adsorption on palladium compared to alkanes, resulting in a higher propylene surface coverage under the same conditions [49,50]. Unsaturated species also have a higher tendency to form oligomers and ring structures compared to alkanes. The removal of butylene from the feed stream results in a gradual flux recovery, but, as seen before, a full recovery would not be reached within a reasonable time scale, e.g., several hours. Even a continuous exposure to a H_2_-containing feed in the absence of butylene over a period of >200 h at 350 °C did not result in a substantial recovery of the H_2_ flux, see Figure 7. Prior to the next exposure, an oxidative treatment was therefore consistently performed in order to recover the initial H_2_ flux. The oxidative treatment at the membrane operating temperature removes surface contaminants from the membrane, and thereby reverses the flux decrease [29,40,51]. In the case of severe coke formation, multiple and/or longer treatments in air were found to be required (as shown in, e.g., Figure 5 after coke formation exposing the membrane to a feed gas at a low H_2_ to butylene ratio). Figure 6 summarizes obtained H_2_ flux inhibition curves as a function of the H_2_/butylene ratio at 400 °C. The results were plotted as a relative flux to allow for a direct comparison of the propene inhibition and coke formation effect obtained at the different ratios. 

It is shown that the poisoning effect of butylene generally increases with a decreasing H_2_/butylene ratio. It should also be noted that the rate of flux recovery in the absence of butylene is increasing with the H_2_/butylene ratio of the performed exposure, which is probably related to the amount of carbon formed during the experiment. This agrees with previous results on propylene [39,40]. In a membrane-enhanced dehydrogenation process, however, at realistic conditions of hydrogen recovery, H_2_/butylene ratios of 0.33–0.5 are typically encountered [6,23,41]. From Figure 6 it can be seen that at the H_2_/butylene ratio of 0.5, the H_2_ flux decreases by approximately 95% after three hours of exposure, which would not be acceptable from a process point of view. It would thus be required to decrease the membrane temperature in order to prevent coke formation in the presence of butylene. Alternatively, a protective coating can be applied on top of the Pd-based membrane surface to prevent the detrimental gaseous components that are responsible for the flux decrease from reaching the Pd membrane surface [52,53], but this was not part of the current study. 

**Figure 8:**
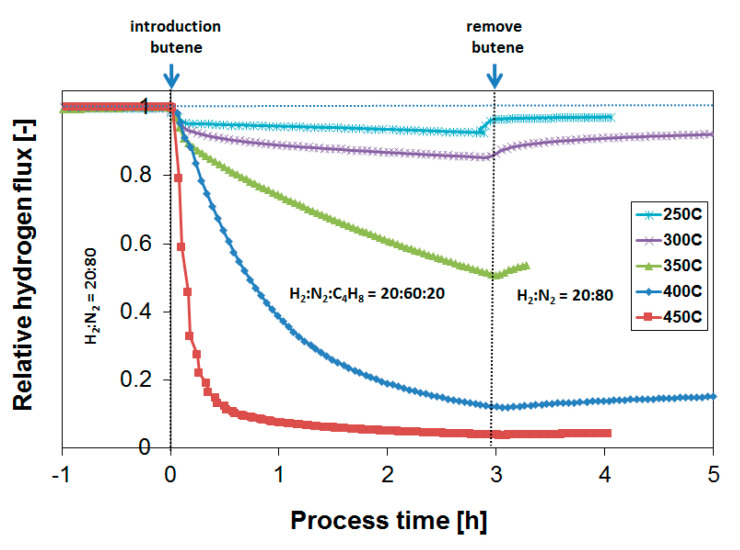
Effect of temperature on the obtained relative H_2_ flux after butylene introduction. The H_2_/butylene ratio is equal to 1.

Subsequently, coke formation kinetics were investigated as a function of temperature between 250 and 450 °C at a fixed H_2_/butylene ratio of 1. Figure 7 shows the H_2_ flux during butylene exposure to a feed composition of H_2_:N_2_:C_4_H_8_ = 20:60:20 at 350 °C, while Figure 8 summarizes the obtained H_2_ flux inhibition curves as a function of temperature, again plotted in relative flux numbers.

Figure 8 shows that the extent of coking decreases rapidly with temperature, and coke formation is rather limited already at 250 °C under the conditions investigated, leading to stable membrane operation. This is as expected from similar studies with respect to the development of dehydrogenation catalysts [54]. It is thus potentially possible, in a non-integrated sequential reactor/separator process scheme, to operate the membrane module at a lower temperature than the dehydrogenation reactor, thereby allowing for stable membrane operation. In terms of the absolute attainable H_2_ flux, the negative effect of this temperature decrease is very limited due to the low activation energy of permeation. Moreover, the produced H_2_ might even be applied to heat the incoming feed stream to the next dehydrogenation reactor. However, to investigate whether these operating conditions allow for sufficiently stable membrane operation, which preferably matches the lifetime of the catalyst system, long-term exposures were investigated.

### 3.4. Long-Term Performance During Butylene Exposure

**Figure 9:**
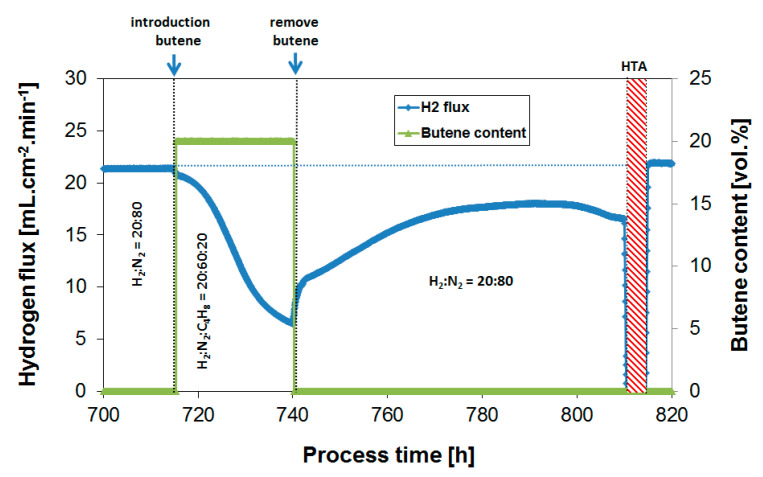
H_2_ flux during prolonged exposure to butylene (20%) in a feed containing: H_2_:N_2_:C_4_H_10_ = 20:60:20. Subsequent air treatment to regenerate the membrane performance. Temperature = 250 °C.

The behavior of membrane performance over prolonged exposure to butylene (24 h instead of 3 h) at 250 °C is reported in Figure 9. Even though coke formation in the short term is rather limited already at 250 °C (Figure 8), the prolonged exposure to the same temperature leads to drastically reduced H_2_ fluxes. After 24 h, the H_2_ flux decreased to a value of approximately 30% of the original H_2_ flux.

**Figure 10:**
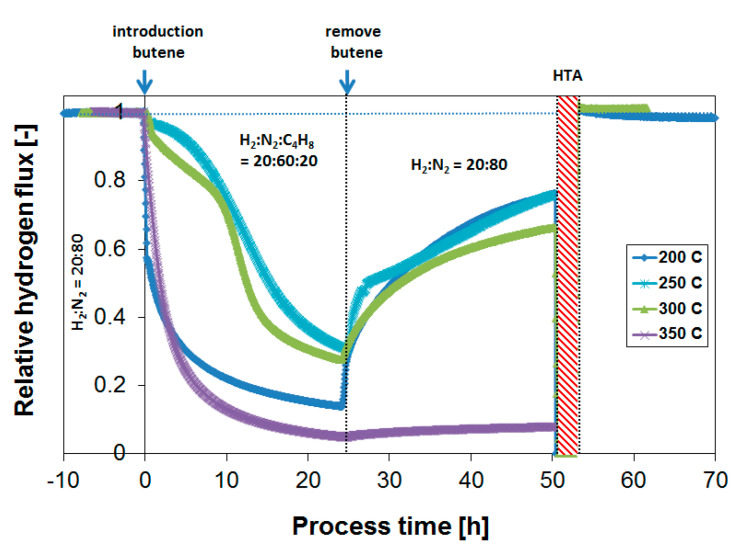
Effect of temperature on the obtained relative H_2_ flux during prolonged butylene exposure. H_2_:N_2_:C_4_H_10_ = 20:60:20.

Figure 10 summarizes obtained H_2_ flux inhibition curves as a function of temperature plotted as relative flux numbers. A further decrease to 200 °C did not improve the membrane operability. On the contrary, even though coke formation tendency in itself was lowered with temperature, the competitive adsorption of butylene with respect to hydrogen accounted for a large initial flux penalty. This is visualized by the large immediate flux decrease directly after butylene introduction for the exposure at 200 °C. A similar initial flux decrease due to absorption is not visible at higher temperatures. 

In terms of operating temperature, an optimal temperature was thus found at 250–300 °C with respect to obtaining the highest absolute hydrogen flux in the presence of butylene. The same can be concluded from the flux recovery in the absence of any butylene. Whereas the flux decrease that occurred during the exposure at 350 °C was persistent, the flux decrease due to the surface C_x_H_y_ species or coke formed at lower temperatures was recovered at a higher rate. This suggests that more persistent poisoning species form at temperatures > 300 °C, which may include various surface species and subsurface carbon [40]. 

### 3.5. Membrane Stability

#### 3.5.1. Separation Performance

**Figure 11:**
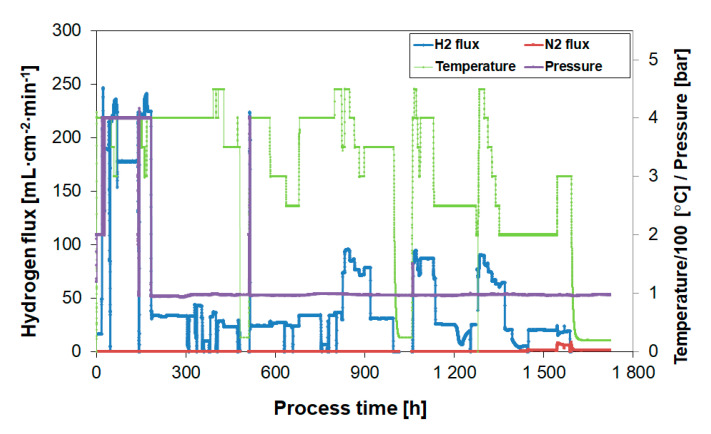
Membrane module #1; 1600 h operation up to 450 °C and 4 bars.

Figure 11 shows the performance of membrane module 1 during nearly 1600 h of operation during which the module was operated up to 450 °C and 4 bars, respectively. During the operation, the module went through two start-up and shut-down cycles and was exposed to air at elevated temperatures for a total of 15 times to regenerate the performance after hydrocarbon exposure.

During the initial 1500 h of testing, no unselective flow of N_2_ was observed, showing that the membrane was 100% selective to H_2_. However, after approximately 1600 h, during the evaluation of the butylene effect at 200 °C, the membrane started to leak, as indicated by the appearance of N_2_ on the permeate side of the membrane. The module was then cooled down and made available for post-process characterization. A new membrane module was prepared for the longer-term exposure experiments to butylene as described in Section 3.4. This second module was operated for nearly 1000 h and kept 100% selective to hydrogen throughout the test.

#### 3.5.2. Post-Process Membrane Characterization

**Figure 12:**
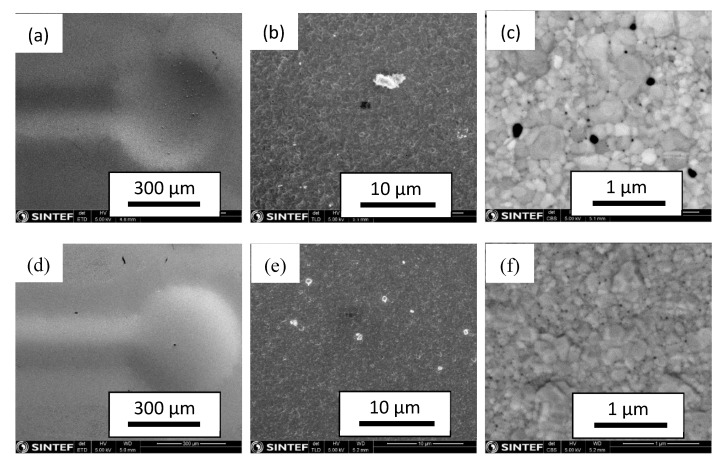
SEM images of the Pd_77_Ag_23_ film after testing: (**a**–**c**) feed (growth side) and (**d**–**f**) permeate (silicon side), at different magnifications.

SEM micrographs of the feed and permeate surface of the tested membrane (module #1) are shown in Figure 12. Figure 12a,d clearly shows the imprint of microchannels across the Pd_77_Ag_23_ film. This is due to plastic deformation of the thin film into the channel support during the higher-pressure operation, as previously reported in [55].

After 1600 h of operation, the feed and permeate sides had a fairly similar grain size, approximately 200–400 nm, which is different from the initial untreated membrane with much smaller grains at the permeate side [46]. Grains with a similar size were observed after long-term operation of a Pd_77_Ag_23_ membrane in the absence of any butylene, though over a period of 100 days at up to 20 bar and 450 °C [56]. Thus, the grain growth cannot be clearly linked to the butylene exposure and is most probably simply linked to the high-temperature operation of these types of membranes [57]. An analysis of the two respective sides of the membrane by energy dispersive X-ray spectroscopy (EDS) shows a higher level of carbon on the feed side of the membrane, supporting previous results concluding that the gradual decrease in H_2_ flux during alkene exposure is due to coke formation on the membrane surface.

Figure 12c,f show as well that pinholes have developed over the long-term testing of the membrane. The development of pinholes is likely to be linked to structural changes resulting from temperature, strain and chemical potential gradients, grain growth and grain boundary deformation in particular. The largest pinholes have a diameter of approximately 50–100 nm, but both Figure 12c,f show a high density of much smaller pinholes that are primarily located at the grain boundaries. Previous work and on-going research show that Pd-based membranes develop cavities along the grain boundaries [58,59,60]. These cavities may represent initial stages of pinhole formation, which lead to unselective leakage and compromise the long-term stability of the membranes. It should also be noted that the current film went through 15 exposures to air at high temperatures to regenerate the performance after hydrocarbon exposure. As shown previously [51,61,62], air-treated membranes can develop structural changes, i.e., void formation and surface roughening, during an oxidation–reduction cycle. For the current membranes, it can thus also be that the pinhole formation is simply related to the multiple heat treatment procedures in air, even though other processes generating defects cannot be ruled out.

## 4. Conclusions

The hydrogen flux characteristics of a Pd–Ag membrane were evaluated for hydrogen/butane/butylene feed gas mixtures. We report the effect of a wide range of operating conditions, such as temperature (200–450 °C) and H_2_/butylene (or butane) ratio (0.5–3), on coke formation kinetics. In the presence of butane, the flux-reducing tendency was found to be limited up to the maximum temperature investigated, 450 °C. Compared to butane, the flux-reducing tendency in the presence of butylene was severe. At 400 °C and 20% butylene, the flux decreases by ~85% after 3 h of exposure, but it is shown that the decrease depends on temperature and the H_2_/butylene ratio. In terms of operating temperature, an optimal temperature was found at 250–300 °C with respect to obtaining the highest absolute hydrogen flux in the presence of butylene. At lower temperatures, the competitive adsorption of butylene over hydrogen accounts for a large initial flux penalty. Whereas the flux decrease that occurred during the exposure at 350 °C was persistent, the flux decrease observed at lower temperatures was recovered at a higher rate. This suggests that more persistent poisoning species form at temperatures > 300 °C, which may include various surface species and subsurface carbon. The experimental sequence was performed applying two membrane modules over a period of 110 days (~2500 h) during which the membranes were operated up to temperature of 450 °C, and pressure up to 4 bars. A tendency of nano-size pore formation was observed by post-process SEM characterization. 

## Figures and Tables

**Table 1:** Hydrogen flux and permeability as a function of operation temperature. A membrane thickness of 10 µm was used to calculate the permeability.

Operation Temperature [°C]	H_2_ Flux [mL·cm^−2^·min^−1^]	H_2_ Permeability [mol·m^−1^·s^−1^·Pa^−0.5^]
450	95.4	3.0·10^−8^
400	86.4	2.7·10^−8^
350	78.4	2.4·10^−8^
300	71.7	2.2·10^−8^
